# Effects of *Lactiplantibacillus plantarum* DSM 33464 in children with elevated blood lead levels: a randomized, double-blind, placebo-controlled study

**DOI:** 10.3389/fnut.2025.1641839

**Published:** 2025-09-01

**Authors:** Wenjing Ji, Delphine Marie Saulnier, Lan Zhang, Jingxia Liu, Jisheng Gao, Xia Wang, Caterina Holz, Aimin Liang, Hern-Tze Tina Tan

**Affiliations:** ^1^Department of Children’s Health Care Center, Beijing Children’s Hospital, Capital Medical University, National Center for Children’s Health, Beijing, China; ^2^Novozymes Berlin GmbH, Berlin, Germany; ^3^Chengdu Women's and Children's Central Hospital, School of Medicine, University of Electronic Science and Technology of China, Chengdu, China; ^4^Baoding Children's Hospital, Baoding, China; ^5^Xuzhou Children’s Hospital, Xuzhou, China; ^6^Children’s Hospital of Hebei Province, Hebei, China; ^7^Novonesis, Cork, Ireland

**Keywords:** probiotic, children, blood lead levels, abdominal pain, developmental health, multivitamin and mineral supplement

## Abstract

**Introduction:**

Approximately one-third of the world’s children have elevated blood lead levels (BLLs), which may lead to often-irreversible decreased intelligence, behavioral difficulties, and learning problems. Identification and removal of the source of lead, along with good nutrition, are the only advocated initial management, with chelation therapy for higher threshold BLLs. Probiotics have shown promising beneficial effects pre-clinically. Here, we investigated the safety and efficacy of the probiotic *Lactiplantibacillus plantarum* DSM 33464 in children with elevated blood lead levels.

**Methods:**

Children aged 3–12 years with elevated BLLs (>3.5 μg/dL) were enrolled in a randomized double-blind placebo-controlled multi-centered study and received either probiotic (1 × 10^9^) colony-forming units (CFUs) or placebo (control group), along with a multivitamin/mineral supplement in both groups daily for 12 weeks.

**Results:**

Overall, 66 children were randomized, 54 received intervention (probiotic; *n* = 30 and control; *n* = 24). The probiotic was well-tolerated. Probiotics, combined with a multivitamin/mineral supplement, significantly reduced the BLLs in these children within 12 weeks of supplementation by 40%, similar to that of the control group, which received a placebo plus multivitamin/mineral supplement. A larger reduction in urine lead levels at 8 weeks was observed in the probiotic group, along with a reduction of abdominal pain and psychosomatic feelings at week 12. No depletion of essential minerals was observed in any of the groups.

**Conclusion:**

This study adds to previous findings suggesting that probiotic intervention may be a promising additional strategy to help reduce BLLs and their detrimental effects in children. Due to the preliminary nature of this study, larger studies investigating the effects of the strain alone, with a longer intervention period, are warranted to confirm the benefits observed.

**Clinical trial registration:**

Clinicaltrials.gov, identifier NCT04891666.

## 1 Introduction

Lead exposure is a worldwide health threat with massive impacts but little awareness, with plenty of existing or even growing sources of exposure, especially in low- and middle-income countries (LMICs) (1, 2). The adverse effects of lead on health have been well documented (3), with cognitive deficits in children even at the lowest blood lead levels (BLLs) (≤5 μg/dL) being the most substantiated effects, where the decrements in intelligence quotient increase with BLLs (4) and are sustained over time.

At low to moderate levels of exposure, there are usually no symptoms, which can impede detection (2). There is no safe level of lead in blood; however, approximately one-third of children (800 million) globally have elevated BLLs at or above 5 μg/dL. In the US, the Center for Disease Control and Prevention has updated the blood lead reference value (BLRV) to 3.5 μg/dL to identify children <6 years with BLLs higher than majority of the children’s levels (5), while the BLRV for children in China remains at 10 μg/dL since 2006 (6).

In China, although the BLLs in children have declined fast over time since phasing out leaded gasoline from 2000, the average BLLs in children of 5.97 μg/dL are still higher than in the USA and Europe, with greater loss of disability adjusted life years than reported for other LMICs (7).

The largest source of lead exposure in China was water pollution (76.16%), followed by air and soil pollution (8), while the main dietary sources of lead were cereals (43.5%), vegetables (29.0%), and beverages and water (9.8%) (9). In addition to the respiratory tract, lead is predominantly absorbed via the gastrointestinal route of exposure, where children absorb approximately 50% of ingested lead after a meal and up to 100% on an empty stomach (10). Biomonitoring data from 2004 to 2014 showed an estimated daily lead exposure through diet in urban children (0–7 years) in China of 12.01 ± 6.27 μg/day (11). As children are more susceptible to lead than adults due to their developing systems, higher absorption, and behaviors that increase ingestion of surface dust (12, 13), solutions targeted at reducing lead absorption, in addition to strategies that prevent exposures, are necessary.

Among the dietary interventions studied for reducing the effects of lead exposure, probiotics have emerged in recent years as promising candidates (14, 15), as there is a mutual and bidirectional relationship between the gut microbiota and lead exposure. Lead exposure alters the microbiome composition and metabolomic profiles of the gut microbiota, which, in turn, is capable of metabolizing and limiting lead absorption, as well as promoting excretion (16–18).

The strain *Lactiplantibacillus plantarum* DSM 33464 (also known as *L. plantarum* CCFM 8610 or SmartGuard™), isolated from a Chinese plant, has shown strong ability to retain and sequester lead in simulated gastrointestinal condition, and has shown promising results of reduced dietary lead absorption in the gastrointestinal tract, leading to reduction in blood, urine, and organs such as kidney and liver, in mice models (unpublished reports: Jiangnan University, The protective effects of probiotics on lead detoxification *in vivo*, 2018; Nanna Ny Kristensen, Acute lead challenge model, 2019). Here, we report findings from a study that assessed the safety and tolerability of this strain and its efficacy in reducing BLLs in Chinese children aged 3–12 years with elevated BLLs.

## 2 Materials and methods

### 2.1 Lead binding assay and transmission electron microscopy of *Lactiplantibacillus plantarum* DSM 33464

Prior to the production of investigational products, the lead-binding capacity of the strain was investigated. First, 100 mg of freeze-dried powder was dissolved in 10 mL ultra-pure water, vortexed briefly, and allowed to rehydrate for 15 min following pH adjustment to 6. Lead-acetate solution (1.45 mM) was then incubated with the solution containing the rehydrated strain for 1 h at 37°C while shaking in a ThermoMixer comfort (150 rpm) (Eppendorf, Germany) to allow binding activity. After centrifugation (10 min, 4,500 *g*), the appropriate amount of pellet sample was placed in a fixative solution (2% paraformaldehyde + 2.5% glutaraldehyde) for 1 h and then rinsed with 0.1 M phosphate-buffered saline (PBS). The samples were further fixed in an osmium tetroxide solution (1% osmium tetroxide + 1.5% potassium ferrocyanide) for 1 h and dehydrated with a graded ethanol series (30, 50, 70, 80, 90, and 100% ethanol), followed by propylene oxide. After embedding in resin, the samples were further prepared into 70-nm sections by Leica EM UC7 (Leica Microsystems, Germany) for transmission electron microscopy observation (Hitachi H7650B, acceleration voltage 80 kV) (Hitachi High-Technologies Corporation, Japan).

### 2.2 Study design and participants

This randomized, double-blind, placebo-controlled, parallel-group, multi-center study was conducted from July 2021 to March 2023 at five clinical sites in China: Beijing Children’s Hospital/Capital Medical University, Baoding Children’s Hospital, Xuzhou Children’s Hospital, Chengdu Women’s and Children’s Central Hospital, and Children’s Hospital of Hebei Province (ClinicalTrials.gov registration: NCT04891666). The protocol was approved by the Ethics Committee of Beijing Children’s Hospital, Capital Medical University, on 21 September 2020, with three amendments: Version 1.1.0 (18 December 2020), Version 2.0.0 (6 August 2021), and Version 3.0.0 (21 December 2021). The study was conducted in accordance with the International Conference on Harmonization (ICH) Good Clinical Practice, as well as the Declaration of Helsinki on ethical principles for medical research involving human subjects (ICH 1996), and applicable regulatory requirements.

The study included five clinic visits: visit 1 screening (day–14 to day–2), visit 2 randomization (day–1), visit 3 follow-up (day 28 ± 3; week 4), visit 4 follow-up (day 56 ± 3; week 8), visit 5 (day 84 ± 3; week 12), and visit 6 follow-up phone calls 4 weeks post-intervention (day 112 ± 3; week 16). Healthy subjects who met the following selection criteria participated in this study: aged 3–12 years, with BLLs (3.5–24.9 μg/dL), subjects, their parents or legal guardians were able and willing to comply with research guidance, and subjects’ parents or legally acceptable representatives signed written informed consent. Eligible subjects were randomized into an experimental group (probiotic) or a control group (placebo), both of which received health education and a daily nutrition/multivitamin/mineral supplement from YingKangWei (Beijing Kangyuan Youte Medical Technology Co., Ltd., China) throughout the 12-week intervention period. Additional information on visit assessments and exclusion criteria is available in the Supplementary Material.

The primary objective was to evaluate the efficacy of *L. plantarum* DSM 33464 (probiotic) in children with elevated BLLs. The secondary objective was to evaluate the safety of *L. plantarum* DSM 33464 in children with elevated BLLs.

### 2.3 Investigational products (IPs) and blinding

The experimental group intervention consisted of *L. plantarum* DSM 33464 (probiotic) at a dose of 1 × 10^9^ colony forming units (CFUs) and dextrin in one sachet (2 g) daily, and the multivitamin/mineral supplement YingKangWei (Beijing Kangyuan Youte Medical Technology Co., Ltd., China) (vitamin B1: 1.5 mg; vitamin B2: 0.8 mg; vitamin B3: 5 mg; vitamin B5: 1 mg; vitamin B6: 0.5 mg; vitamin B7: 10 μg; vitamin B9: 80 μg; vitamin B12: 1 μg; vitamin A: 600 IU; vitamin D: 200 IU; iron pyrophosphate: 5 mg; calcium: 180 mg; zinc gluconate: 4 mg; maltodextrin: 1.4 g) one sachet daily for 12 weeks. The control group received one sachet of placebo (dextrin only, 2 g) and the supplement YingKangWei one sachet daily for 12 weeks. Subjects were instructed to take one sachet of IPs (probiotic or placebo) orally 0.5 h before having a meal, by dissolving into a moderate amount of lukewarm water, once daily, and one sachet of YingKangWei orally, by dissolving into moderate amount of warm water or food, with dosing interval of at least 4 h from IP intake. An IP dispensing and return log were used to account for all IPs dispensed and returned.

Permuted block randomization was used to randomize eligible children into two arms with a 1:1 ratio. The subjects, investigators, and all clinical personnel were blinded to intervention arms until the database was locked. No emergency arose throughout the study that necessitated unblinding. The IPs had the same visual appearance to avoid compromising the study blinding. Additional information is available in the Supplementary Material.

### 2.4 Endpoints

The primary endpoint of the study was the difference in change from baseline BLLs between the probiotic group and the control group at week 12. Secondary endpoints included the differences in change from baseline BLLs between groups at weeks 4 and 8; the differences in change from baseline urine lead levels (ULLs) between groups at weeks 4, 8, and 12; the differences in change from baseline improvement of common trace elements (calcium, zinc, copper, magnesium, iron) levels in the blood between groups at weeks 4, 8, and 12. Exploratory endpoints included the changes in total scores and subscores of the 48-item Conners’ Parent Rating Scale (CPRS) (19) that measures children’s emotional and behavioral attitude by including five subscales assessing conduct problem, learning problem, anxiety, impulsive/hyperactive behavior and psychosomatic feelings and Gastrointestinal Symptoms Rating Scale (GSRS) (20, 21) which measures 15 gastrointestinal symptom items by a 4-point scale rated from 0 to 3 (with higher score representing worse symptom), resulting in a total score and five subscore categories: reflux, abdominal pain, indigestion, diarrhea, and constipation; from baseline to week 12 between groups.

### 2.5 Safety assessment

Safety assessments included adverse events (evaluated and graded according to the National Cancer Institute Common Terminology Criteria for Adverse Events v5.0) throughout the study period (visit 1–visit 6), vital signs (temperature, pulse, systolic blood pressure, and diastolic blood pressure), physical examination, 12-lead electrocardiogram, clinical laboratory examination (routine blood test, blood biochemistry) and GSRS. Vital signs, physical examination, and clinical laboratory examination were measured at visits 1, 3, 4, and 5, while a 12-lead electrocardiogram was measured at visit 1 and after completion of intervention at visit 5. GSRS was assessed at visits 2 to 5.

### 2.6 Statistical analyses

Due to the exploratory nature of the study, the sample size (*n* = 124) was not based on formal statistical considerations. Based on the intent-to-treat principle, all randomized subjects who received at least one intervention dose were included in the Full Analysis Set (FAS), which was used to analyze the demographic and baseline characteristics and efficacy assessment. Safety analysis was based on the Safety Set (SS), that is, all subjects who received at least one intervention dose and had available safety assessment data after baseline. A linear mixed-effects model for repeated measures (MMRMs) was applied to primary and secondary endpoints. Two-tailed tests with a specified type I error rate of 0.05 were used. The significance level was set to 0.05. The least squares means (LSM), standard error (SE), LSM difference (LSMD), and 95% two-sided confidence intervals (CIs) and *p*-values were presented. Sensitivity analyses were performed based on the per-protocol set (PPS) and analysis of covariance (ANCOVA) method. *Post hoc* analysis of GSRS used the MMRM model, while Analysis of Covariance (ANCOVA) was used for the CPRS questionnaire. The analyses were performed using Statistical Analysis System (SAS version 9.4). The graphs were constructed using GraphPad Prism version 10.2.2 (GraphPad Software, San Diego, CA, USA). The results presented focus on the FAS population. Additional information is available in the Supplementary Material.

## 3 Results

### 3.1 *Lactiplantibacillus plantarum* DSM 33464 binding lead

**Figure 1:**
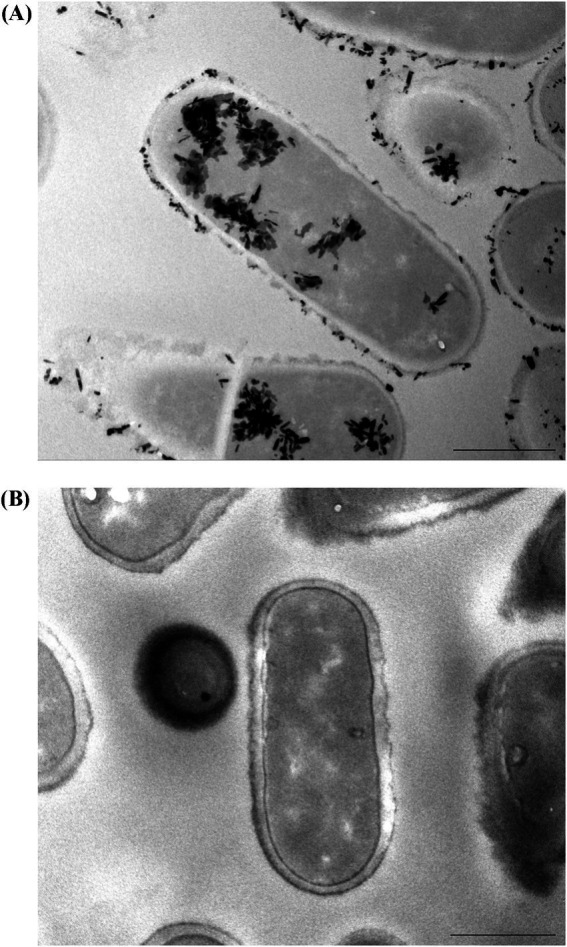
TEM images show **(A)** *in vitro* lead binding and sequestration of *L. plantarum* DSM 33464 and **(B)** *L. plantarum* DSM 33464 in PBS without lead incubation as a control. Scale bar = 500 nm.

The strain used in this trial was shown to have the ability to bind and retain lead *in vitro*, as shown by transmission electron microscopy (Figure 1A) compared to the control (Figure 1B).

### 3.2 Study population

**Figure 2:**
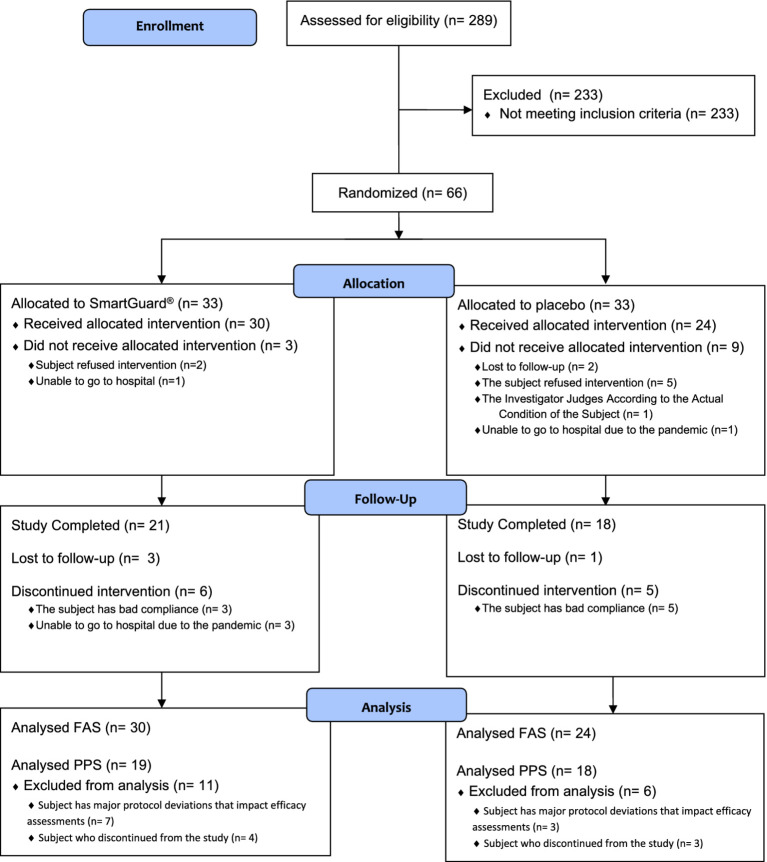
CONSORT flow diagram.

Sixty-six children were randomized (*n* = 33 per arm) (Figure 2). Fifty-four (81.8%) children received at least one intervention and were included in the FAS and SS (probiotic; *n* = 30 and control; *n* = 24). Only 37 (56.1%) children who completed the study and had no major protocol deviations that impacted efficacy assessments were included in the PPS (probiotic; *n* = 19 and control; *n* = 18). The majority (94.4%) of children in FAS had slightly elevated BBL at baseline [median (interquartile range): 4.2 (1.3) μg/dL]. Table 1 summarizes the children’s baseline demographic and physiological characteristics. The data collected showed that those randomized to each group were similar on all demographic and physiological measures (Table 1). Questionnaire on lead exposure sources showed no major differences in both groups (Supplementary Table 1). Interestingly, 53.7% of children overall frequently put things in their mouths or might eat non-food items. The overall IP compliance in the SS was high (probiotic: 90.0%; control: 82.6% subjects with relative dose intensity ≥80% and ≤120%).

**Table 1:** Demographics and physiological characteristics at baseline.

Characteristic	Probiotic		Control	
	*N* = 30 *n* = 6 Female/*n* = 24 Male		*N* = 24 *n* = 8 Female/*n* = 16 Male	
	Mean	SD	Mean	SD
	Anthropometric			
Age (years)	6.9	2.1	6.9	2.2
Weight (kg)	25.9	12.3	26.9	11.3
Height (cm)	123.3	15.2	124.0	17.9
	Vital signs			
Temperature (°C)	36.5	0.2	36.4	0.2
Pulse (beats/min)	84.5	11.7	82.5	17.1
Breathing (breaths/min)	22.6	3.4	24.8	14.1
Systolic blood pressure (mm Hg)	103.0	13.8	99.4	9.8
Diastolic blood pressure (mm Hg)	62.9	8.0	57.6	5.6

Data included in Table 1 represent the FAS population. FAS, full analysis set; N, n, number of subjects by intervention category (N) or sex (*n*); SD, standard deviation.

### 3.3 Efficacy endpoints

**Figure 3:**
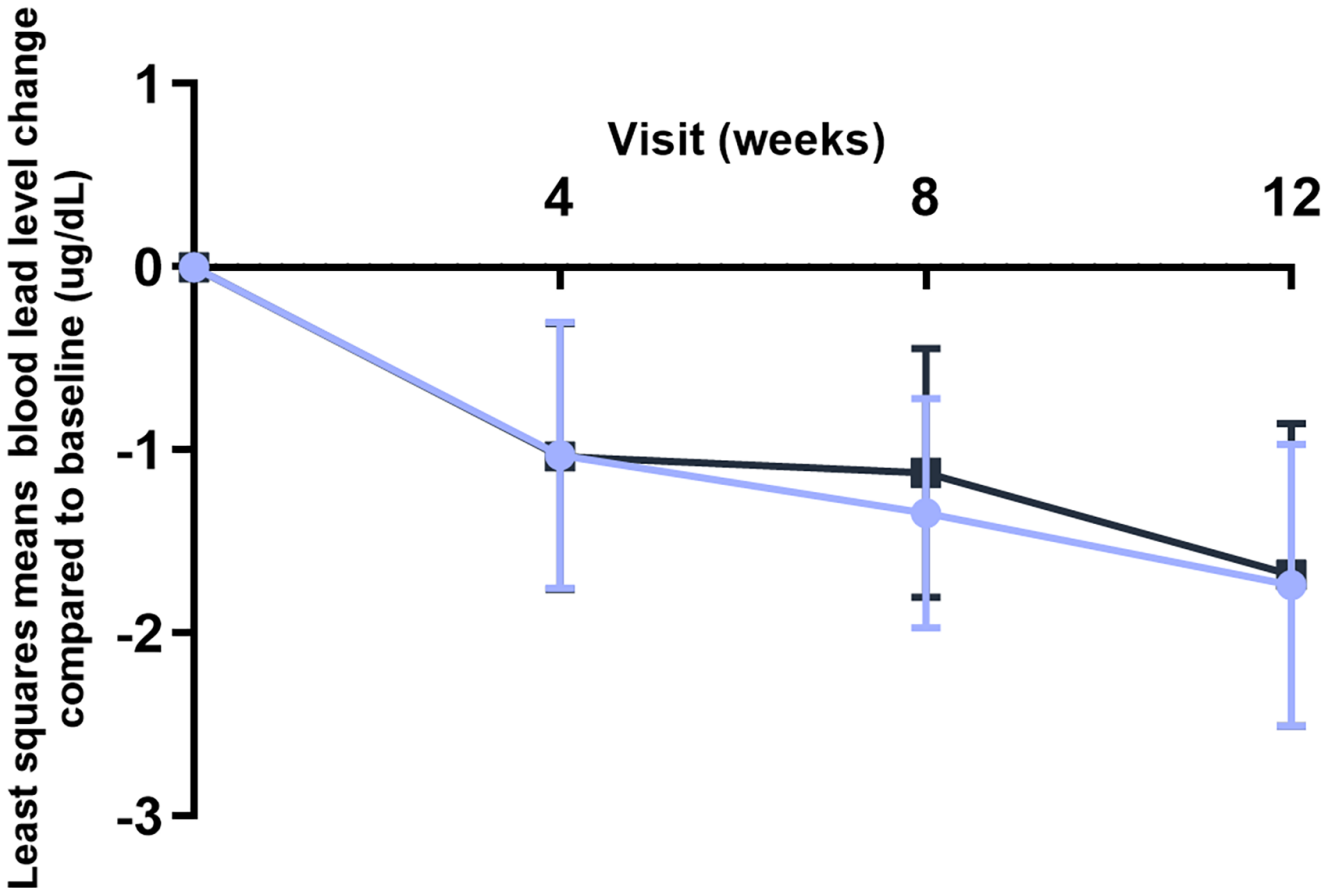
Improvement of blood lead levels (BLLs) over time (FAS population). Values plotted are the least squares means of the changes at weeks 4, 8, and 12 compared to baseline, with 95% confidence intervals. 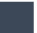 Control. 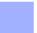 *L. plantarum* DSM 33464.

**Figure 4:**
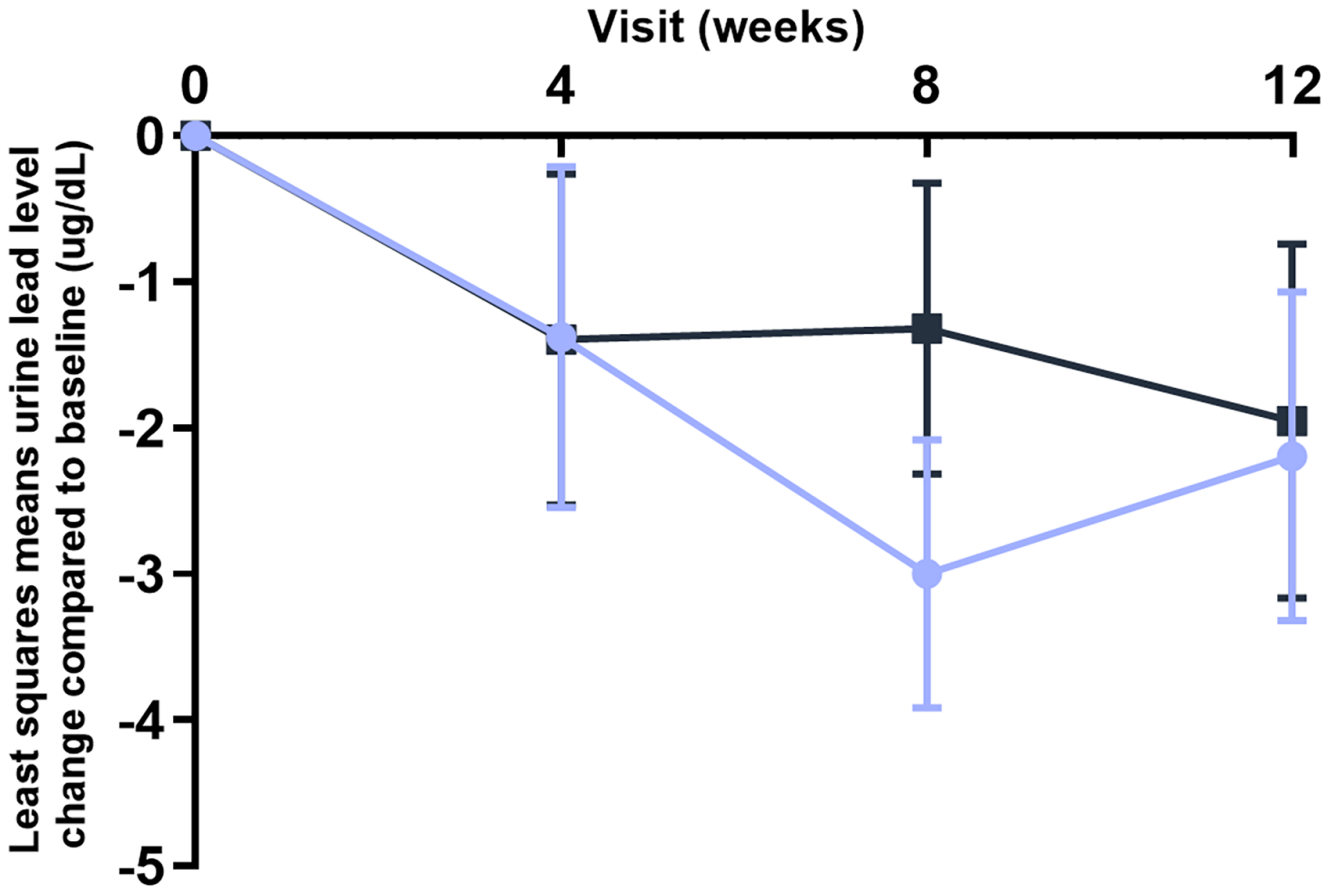
improvement of urine lead levels (ULLs) over time (FAS population). Values plotted are the least squares means of the changes at week 4, 8, and 12 compared to baseline, with 95% confidence intervals. 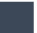 Control. 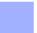 *L. plantarum* DSM 33464.

**Figure 5:**
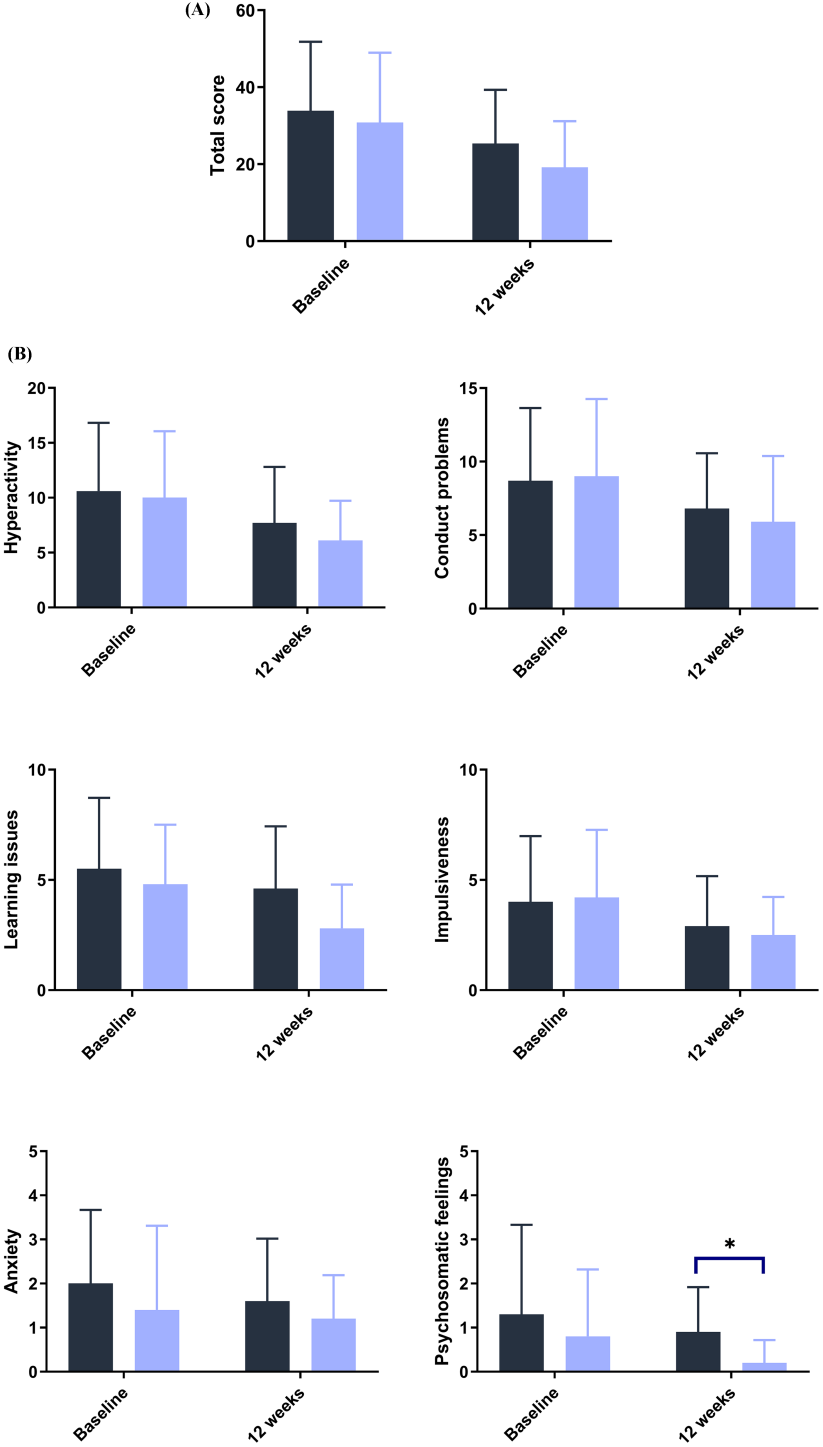
Improvement in emotional behavior as measured by the Conner’s scale score at baseline and 12 weeks **(A)** total and **(B)** subscores. ANCOVA. *Post hoc* analysis. 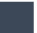 Control. 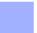 *L. plantarum* DSM 44646. *p< 0.05.

Blood lead levels reduced from baseline to the end of supplementation in both groups by approximately 40% from 5.3 to 3.1 μg/dL in the probiotic and from 4.8 to 2.9 μg/dL in the placebo group, respectively (both *p* < 0.001; Table 2). Probiotic had no further effect on the change in BLLs from baseline to week 4 (*p* = 0.99), week 8 (*p* = 0.64), or week 12 (*p* = 0.92) over that of control [LSMD (95% CI): 0.04 (-10.2, 10.3); -2.2 (-11.5, 7.1); and -0.6 (-11.9, 10.8) for each timepoint respectively] (Figure 3). The results from PPS were consistent with those from FAS [*p*-values for LSMD >0.05 for all timepoints]. Urine lead levels were also reduced from baseline in both groups throughout the study (Figure 4). At week 8, the ULL LSM (SE) for probiotic was -30.0 (4.6) while control was -13.2 (5.0) [LSMD (95% CI): -16.8 (-30.4, -3.2) μg/L] (*p* = 0.02) (Table 2). There was no statistical difference between groups for change from baseline ULLs at weeks 4 and 12. The blood levels of all essential trace elements (calcium, zinc, copper, magnesium, and iron) at week 4, 8, and 12 stayed within normal physiological ranges, without any deficiencies reported. At the end of the intervention, there was no statistically significant difference between groups for all essential elements. The GSRS total score improved for both groups from baseline to week 12, with the abdominal pain score reduced significantly in the probiotic group compared to the control at week 12 (*p* = 0.03) (Table 3). CPRS total score and all subscores improved for both groups from baseline to week 12, with improvement in psychosomatic feelings (*p* = 0.01) and learning problems (*p* = 0.06) in the probiotic group more pronounced than in the control group at week 12 (Table 4; Figure 5).

**Table 2:** Blood lead levels, urine lead levels, and essential trace elements levels in blood.

Timepoint	Probiotic		Control		
	*N*	Mean (SD)	*N*	Mean (SD)	*p*-value
Blood lead level (μg/dL)					
Baseline	30	5.3 (2.8)	24	4.8 (2.1)	
Week 4	24	3.7 (2.0)	24	3.8 (3.0)	0.99
Week 8	25	3.7 (2.0)	21	3.5 (1.5)	0.64
Week 12	21	3.1 (1.7)	18	2.9 (2.0)	0.92
Urine lead level (μg/dL)					
Baseline	30	6.0 (4.8)	24	7.3 (5.8)	
Week 4	23	4.8 (3.4)	24	4.7 (2.3)	0.98
Week 8	25	3.4 (1.6)	21	4.8 (3.1)	**0.02**
Week 12	21	4.2 (2.1)	18	4.4 (2.9)	0.77
Calcium level in blood (mmol/L)					
Baseline	30	1.8 (0.2)	24	1.7 (0.2)	
Week 4	24	1.7 (0.2)	24	1.7 (0.2)	0.85
Week 8	25	1.7 (0.1)	21	1.7 (0.1)	>0.99
Week 12	21	1.7 (0.2)	18	1.7 (0.1)	0.88
Zinc level in blood (μmol/L)					
Baseline	30	75.7 (13.4)	24	74.3 (11.4)	
Week 4	24	78.7 (11.4)	24	78.1 (8.6)	0.91
Week 8	25	72.9 (8.4)	21	75.1 (9.0)	0.44
Week 12	21	74.4 (9.6)	18	72.5 (5.9)	0.52
Copper level in blood (μmol/L)					
Baseline	30	18.8 (2.7)	24	17.5 (2.7)	
Week 4	24	18.9 (2.7)	24	17.4 (2.4)	**0.05**
Week 8	25	17.5 (2.7)	21	17.1 (3.3)	0.57
Week 12	21	17.9 (3.4)	18	19.3 (3.0)	0.20
Magnesium level in blood (mmol/L)					
Baseline	30	1.4 (0.1)	24	1.4 (0.1)	
Week 4	24	1.4 (0.1)	24	1.5 (0.1)	0.10
Week 8	25	1.4 (0.1)	21	1.4 (0.1)	0.60
Week 12	21	1.4 (0.1)	18	1.4 (0.1)	0.43
Iron level in blood (mmol/L)					
Baseline	30	7.6 (0.8)	24	7.6 (0.8)	
Week 4	24	7.8 (0.5)	24	7.7 (0.5)	0.85
Week 8	25	7.6 (0.4)	21	7.9 (0.5)	**0.04**
Week 12	21	7.7 (0.6)	18	7.7 (0.4)	0.92

FAS population. *p*-values were derived from the difference for least squares means, in a linear mixed-effects model for repeated measures (MMRMs) that compared changes with baseline between the experimental group and the placebo group. N, number of subjects; SD, standard deviation. Bold p-value represents statistical significance (*p*< 0.05).

**Table 3:** Gastrointestinal Symptom Rating Scale (GSRS) questionnaire scores.

Timepoint	Probiotic		Control			
	*N*	Mean (SD)	*N*	Mean (SD)	OR (95% CI)	*P*-value
Total score						
Baseline	30	2.0 (2.7)	24	1.4 (2.6)		
Week 4	26	0.9 (1.7)	24	1.1 (1.8)	1.3 (0.2 to 7.0)	0.74
Week 8	25	0.6 (1.7)	21	1.0 (1.9)	1.0 (0.2 to 6.1)	>0.99
Week 12	21	1.1 (2.7)	18	1.3 (1.9)	0.5 (0.1 to 3.1)	0.44
Reflux score						
Baseline	30	0.0 (0.2)	24	0.1 (0.3)		
Week 4	26	0.0 (0.0)	24	0.0 (0.0)	1.3 (0.0 to 73.2)	0.90
Week 8	25	0.1 (0.4)	21	0.0 (0.0)	7.3 (0.14 to 381.8)	0.32
Week 12	21	0.1 (0.4)	18	0.0 (0.0)	12.1 (0.1 to 1096.7)	0.27
Abdominal pain score						
Baseline	30	0.3 (0.5)	24	0.3 (0.6)		
Week 4	26	0.3 (0.6)	24	0.3 (0.6)	2.5 (0.3 to 21.1)	0.40
Week 8	25	0.2 (0.6)	21	0.2 (0.4)	0.8 (0.1 to 9.7)	0.85
Week 12	21	0.1 (0.3)	18	0.4 (0.8)	0.04 (0.0 to 0.7)	**0.03**
Indigestion score						
Baseline	30	0.5 (0.9)	24	0.2 (0.6)		
Week 4	26	0.2 (0.5)	24	0.4 (0.8)	0.3 (0.0 to 1.7)	0.17
Week 8	25	0.1 (0.3)	21	0.1 (0.5)	0.7 (0.1 to 5.1)	0.72
Week 12	21	0.3 (0.8)	18	0.2 (0.4)	1.0 (0.1 to 7.2)	0.97
Diarrhea score						
Baseline	30	0.3 (0.5)	24	0.3 (0.9)		
Week 4	26	0.0 (0.2)	24	0.2 (0.5)	0.1 (0.0 to 1.7)	0.11
Week 8	25	0.1 (0.3)	21	0.1 (0.4)	0.2 (0.0 to 3.5)	0.27
Week 12	21	0.1 (0.3)	18	0.2 (0.6)	0.1 (0.0 to 2.9)	0.20
Constipation score						
Baseline	30	0.9 (1.6)	24	0.5 (1.0)		
Week 4	26	0.3 (0.9)	24	0.3 (0.7)	0.8 (0.1 to 4.7)	0.83
Week 8	25	0.2 (0.7)	21	0.5 (1.1)	0.7 (0.1 to 4.8)	0.74
Week 12	21	0.5 (1.1)	18	0.4 (1.0)	2.8 (0.4 to 21.6)	0.33

Safety set population. *p*-values were derived from a generalized linear mixed model for the probability of having a worse value for change in each GSRS category. N, number of subjects; SD, standard deviation; OR, odds ratio; CI, confidence interval. Bold *p*-value represents statistical significance (*p*< 0.05).

OR<1: Exposure associated with lower odds of outcome.

**Table 4:** 48-item Conners’ Parent Rating Scale (CPRS).

Timepoint	Probiotic		Control			
	*N*	Mean (SD)	*N*	Mean (SD)	Estimate (95% CI)	*p*-value
Total score						
Baseline	30	30.8 (18.2)	24	33.9 (17.9)		
Week 12	20	19.2 (12.0)	18	25.4 (13.9)	−2.7 (−9.2–3.9)	0.41
Conduct problems						
Baseline	30	9.0 (5.3)	24	8.7 (4.9)		
Week 12	20	5.9 (4.5)	18	6.8 (3.8)	−0.7 (−2.9–1.4321)	0.50
Learning problems						
Baseline	30	4.8 (2.7)	24	5.5 (3.2)		
Week 12	20	2.8 (2.0)	18	4.6 (2.8)	−1.1 (−2.1–0.0)	0.06
Psychosomatic feelings						
Baseline	30	0.8 (1.5)	24	1.3 (2.0)		
Week 12	20	0.2 (0.5)	18	0.9 (1.0)	−0.7 (−1.3 – −0.1)	**0.01**
Impulsiveness/hyperactivity						
Baseline	30	4.2 (3.1)	24	4.0 (3.0)		
Week 12	20	2.5 (1.7)	18	2.9 (2.3)	−0.3 (−1.4–0.8)	0.55
Anxiety						
Baseline	30	1.4 (1.9)	24	2.0 (1.7)		
Week 12	20	1.2 (1.0)	18	1.6 (1.4)	0.1 (−0.7–0.8)	0.84
Hyperactivity index						
Baseline	30	10.0 (6.1)	24	10.6 (6.2)		
Week 12	20	6.1 (3.6)	18	7.7 (5.1)	−0.7 (−2.7–1.3)	0.48

Safety Set population. *p*-values were derived from ANCOVA for each CPRS category. N, number of subjects; SD, standard deviation; CI, confidence interval. Bold *p*-value represents statistical significance (*p*< 0.05).

### 3.4 Safety endpoints

The incidence and severity of adverse events were similar in both groups (Supplementary Table 2). There were no treatment-related adverse events (TRAEs), no treatment-emergent adverse events (TEAEs) leading to the investigational product being permanently discontinued or study discontinuation, and no grade 4 or above TEAEs. TEAEs are detailed in Supplementary Table 3.

Routine blood test and blood biochemistry revealed no major findings. No major differences were noted between the groups for all vital signs (Supplementary Table 4).

## 4 Discussion

Prior to clinical intervention, we confirmed the ability of live *L. plantarum* DSM 33464 to bind and retain lead via electron microscopy after a lead-binding assay. The results gave reassurance that the freeze-drying process did not impair the strain’s capacity to bind lead. We then examined the safety and efficacy of *L. plantarum* DSM 33464 in children with elevated BLLs in a randomized, placebo-controlled multi-center study with a 12-week intervention period. For the primary outcome, we found no statistically significant difference between intervention groups, although both groups showed a reduction of BLLs by 40% compared to baseline. For secondary outcomes, probiotics reduced ULLs beyond the control at week 8, while other timepoints showed no statistically significant difference. Overall, the strain was safe and well-tolerated, did not deplete minerals, and significantly improved the abdominal pain score and psychosomatic feelings compared to the control at week 12.

With a median BLL of <5 μg/dL, the children were characterized by having slightly elevated BLLs rather than hyperlipemia according to the Chinese guidelines (22). There are recommendations from the World Health Organization for the management of lead exposure for BLLs ≥ 5 μg/dL, including nutritional supplementation with calcium and iron in case of deficiency, but not for BLLs <5 μg/dL (23). Even so, at this low level, children, especially those younger than 5 years, are most susceptible to the often-irreversible adverse effects of lead, including behavioral and learning problems, lower intelligence quotient, hyperactivity, and impaired growth (2).

Our study is the first to demonstrate BLL reduction with probiotic intervention in addition to multivitamin/mineral supplements in children with elevated BLLs. Two studies have already investigated the effects of probiotics in lead-exposed subjects; one with yogurt containing 10^10^ CFUs of *Lacticaseibacillus rhamnosus* GR-1 that showed no improvement in BLLs in children and pregnant women in Tanzania (24), while another found beneficial effects when *L. plantarum* CCFM8661 with prebiotic was given to lead-exposed adults, by modulating the gut microbiota, and both the metabolism of the host and gut microbiota (25).

A major limitation of our study is that both groups were given multivitamin/mineral supplements including calcium, iron, zinc, and several vitamins on top of the probiotic product or placebo, which may have complemented the effects of the probiotic group. Iron, zinc, calcium, and vitamin C levels and supplementation can influence the gastrointestinal absorption and distribution of lead (26, 27). Diets low in calcium have been reported to enhance lead accumulation (28) while iron-deficient children have higher gastrointestinal absorption of lead (29). Although no specific history of mineral or vitamin deficiency was present in the majority of the children enrolled, this supplementation is a standard care for children with high BLLs at the study site in Beijing, and therefore given to all participating children. There is insufficient evidence on the effects of calcium or iron supplementation on BLLs and clinical outcomes (30), but the observations of significant BLL reduction with calcium supplementation in children and pregnant women with slightly elevated BLLs (31, 32) indicate that the standard care administered likely contributed to the positive outcomes in both groups. Therefore, the lack of a difference in BLLs between the two groups may be attributed to the additional supplementation of multivitamins and minerals given to all children. Furthermore, as the measure of change from baseline BLLs is dependent on the baseline values, the high baseline variability (see Table 2) may have contributed to the lack of difference observed between groups. The other limitations include the effects of the coronavirus disease 2019 (COVID-19) pandemic, such as frequent or constant lockdowns and increased hand-washing, which could have reduced exposure and contributed to improved BLLs and other outcomes measured. The pandemic also delayed and reduced the number of children enrolled (from 124 to 66) and affected protocol compliance. The planned intervention period was also halved from 24 weeks due to the reluctance to participate during this challenging period.

We found a significantly larger reduction in ULLs with probiotics compared to the control at week 8, and a trend at week 12 that was no longer statistically significant, possibly due to the reduced samples available and large inter-individual variation (33). Urine lead, which reflects recent absorption (33), has been used for monitoring only to some extent (34, 35) as >99% of lead in blood is found in erythrocytes, while urine lead originates from plasma (36). As urine and feces are the two main pathways of lead excretion from the body, a decrease in urine lead excretion could reflect reduced absorption in the gastrointestinal tract and/or more excretion through the fecal route, in parallel with reduced BLLs observed. Fecal lead was not studied as the analytical methods are not validated nor available in the central laboratory. The ULLs in our study were not adjusted for diuresis using creatinine, which would have made it more reliable, as creatinine-corrected urine may account for variations by normalizing individual differences in hydration and kidney function, reflecting the lead burden in the body more accurately (37, 38). Therefore, creatinine excretion correction for ULLs is recommended for future studies (39).

There are multiple mechanisms described for how probiotics may remove heavy metals (40), while *L. plantarum* DSM 33464 showed, in addition to heavy metal binding and sequestration ability, strengthening of the gut barrier (41, 42). In addition, the regulation of bile acid enterohepatic circulation, which is related to gut microbiota remodeling, has been found in cadmium-exposed adults after 8 weeks of supplementation with the strain, resulting in significantly reduced blood cadmium levels compared to placebo (26). This strain did not show any TRAEs in adults when previously studied in four studies with different indications (26, 43–45). We have now confirmed that it is also well-tolerated in children.

The positive effects of the probiotic group over the control group on abdominal pain and psychosomatic feelings are encouraging, even with complementary effects from multivitamin/mineral supplementation, and potential reduced exposure due to the pandemic and education. Although few children reported only minor gastrointestinal symptoms at baseline, they improved over time in the probiotic group. These results are in line with previously demonstrated symptoms’ improvements in irritable bowel syndrome patients after 8 weeks’ intervention with the strain (43). The observed improvement in psychosomatic symptoms could be plausibly linked to reduced abdominal pain.

## 5 Conclusion

*L. plantarum* DSM 33464, combined with a multivitamin/mineral supplement, reduced BLLs in children with elevated BLLs within 12 weeks, and improved associated abdominal pain and psychosomatic feelings; therefore, it is a promising, safe intervention for children with slightly elevated BLLs. Larger studies with the strain alone, featuring a longer intervention period, and more thorough lead monitoring are warranted to confirm the effects of the strain.

## Data Availability

The raw data supporting the conclusions of this article will be made available by the authors upon reasonable request.
