# Supplementary material for: Effects of Lactiplantibacillus plantarum DSM 33464 in children with elevated blood lead levels: a randomized, double-blind, placebo-controlled study
Source: Front Nutr. 2025 Sep 1;12:1641839. doi: 10.3389/fnut.2025.1641839 (PMC12434113; doi:10.3389/fnut.2025.1641839)
Supplement: Supplementary file 1 [file Supplementary_file_1.docx]

**Effects of *Lactiplantibacillus plantarum* DSM 33464 in children with elevated blood lead levels: A randomized, double-blind, placebo-controlled study**

Supplemental information

**METHODS:**

**VISIT ASSESSMENTS**

At screening visit (visit 1), subjects underwent eligibility check and various assessments after they or their guardians signed the informed consent form (ICF). Demographic data, medical history, and data from safety assessment [including vital signs, physical examination, routine blood test, blood biochemistry, 12-lead electrocardiogram (ECG)], venous blood lead test, trace element test, urine lead test, questionnaires [Parents’/Guardians’ questionnaires (on family situation, potential lead exposure screening, thoughts related to exposure), Conners’ Parent Rating Scale (CPRS)), concomitant medications and adverse events (AEs) were collected.

At visit 2, subjects’ eligibility based on inclusion and exclusion criteria were checked again before being randomized to start intervention. Gastrointestinal Symptoms Rating Scale (GSRS) questionnaire, concomitant medicine and AEs were recorded.

Subjects had two visits mid-intervention, at week 4 (visit 3) and week 8 (visit 4), and an end of intervention visit at week 12 (visit 5). These three visits comprise of the same assessments for safety (as listed above, except 12-lead ECG which was only done at visit 5) and efficacy evaluation (venous blood lead test, trace element test, urine lead test, GSRS questionnaires). visit 4 had additional data collection on Parents’/Guardians’ questionnaires and Conners’ Scale. Concomitant medicine and AEs were recorded at all these visits.

For the safety of subjects, those who withdrawn/discontinued treatment for any reason had to return to the study center within 28 days after withdrawal/discontinuation for assessments and data collection as required for visit 5.

Subjects who completed the intervention received follow-up phone call at week 16 (visit 6) to collect data on concomitant medication and adverse event (AE).

**EXCLUSION CRITERIA**

Any of the following was regarded as a criterion for exclusion of a participant from the study:

1. diagnosed with nervous system diseases, genetic and metabolic diseases, endocrine diseases, lung diseases, severe or unstable cardiovascular diseases, clinically significant kidney or liver diseases, blood system diseases, any other clinically significant diseases and other investigators’ judgements that the participation of subjects in the study will increase the risk of the subjects' diseases.
2. history of infection or organ transplantation of human immunodeficiency virus or other acquired congenital immunodeficiency diseases.
3. took probiotic products in the last two weeks.
4. have known or suspected sensitivity or allergy to food or any constituents tested in the study.
5. participated in another clinical study or food study 4 weeks prior and during the study.

**RANDOMIZATION**

Permuted Block randomization method was used to generate random codes through the SAS9.4 randomisation process to randomized all subjects into the SmartGuard™ group and the Control group with a 1:1 ratio.

According to the screening order, each subject was assigned a unique screening number. The random table was generated through Interactive Web Response System (IWRS) and assigned unique random numbers and groups to subjects who meet the enrollment criteria in the order of enrollment. The random number was unable to be re-used. Subject replacement was considered when the subject fails to receive the initial scheduled investigational food for reasons other than safety. Substitute subject received the same investigational product as the original subject.

**INVESTIGATIONAL PRODUCT COMPLIANCE CALCULATION**

Number of investigational products and YingKangWei supplements dispensed, used, and returned were recorded, and compliance was calculated from recorded duration of exposure, actual cumulative dose, planned cumulative dose, and relative dose intensity. The relative dose intensity was classified as <80%, 80%-120%, and >120%.

Duration of exposure (days) was calculated as the last date of receiving the investigational product -The first date of receiving the investigational product +1; actual cumulative dose (bags) was the sum of the actual cumulative dose (sachets) at each visit, which equals to (The end date of receiving the investigational product -The start date of receiving the investigational product +1) × actual daily dose (sachets); planned cumulative dose (bags) refers to duration of exposure ×planned daily dose from protocol (sachets); and relative dose intensity (%) was actual cumulative dose (bag)/planned cumulative dose (bag)×100%.

**STATISTICAL ANALYSES**

Linear mixed-effects model for repeated measures (MMRM) were applied to compare the difference in the improvement of BLL compared with baseline between SmartGuard™ group and control group at week 12. The model included subject as a random variable and intervention group, visit (4, 8 and 12 weeks), baseline BLL values, and interaction between intervention group and visit as fixed variable. The restricted maximum likelihood estimation (REML) was used. The within-subject covariance structure was the unstructured covariance. The Kenward-Roger method was used to estimate denominator degrees of freedom. The same analysis methods were applied for all other secondary outcomes.

Per-protocol Set (PPS) consists of all subjects in the FAS who completed the study without major protocol deviations that impact efficacy assessments. Sensitivity analyses were performed based on PPS and ANCOVA method with missing value imputation for primary and secondary outcomes at week 4, 8 and 12 visits. If the outcome was missing at the post-baseline visits and at least one post-baseline measurement was available, the last observation carried forward (LOCF) method was then applied to imputation. The imputation was not applied if all post-baseline assessments were not done. ANCOVA was applied to provide LS means, SE, LSMD and 95% two-sided CIs and p-values for the change from baseline at each post-baseline visit. The baseline value was a covariate in the model.

In post-hoc analysis, to control for the center effect, the center, interaction between center and intervention, and interaction between center, intervention and visit were included in the MMRM as covariates if differences between the SmartGuard^™^ and Control group were observed at week 12 in the MMRM FAS analysis per pseudo-center for BBL and ULL.

The MMRM model was applied to the post-hoc analysis of the GSRS questionnaire. The analysis was performed on the FAS, as well as on the participants on the FAS whose participants had GSRS at baseline. The analysis was performed on the total score, as well as on each of the different subscores.

For the analysis of the CPRS questionnaire, post-hoc analysis using ANCOVA was performed on the FAS, for total score and subscores.
