# Supplementary material for: Effects of Lactiplantibacillus plantarum DSM 33464 in children with elevated blood lead levels: a randomized, double-blind, placebo-controlled study
Source: Front Nutr. 2025 Sep 1;12:1641839. doi: 10.3389/fnut.2025.1641839 (PMC12434113; doi:10.3389/fnut.2025.1641839)
Supplement: Supplementary file 2 [file Supplementary_file_2.docx]

**Effects of *Lactiplantibacillus plantarum* DSM 33464 in children with elevated blood lead levels: A randomized, double-blind, placebo-controlled study**

**Supplemental Tables**

**Supplemental Table 1. Sources of lead exposure**

|  |  | **Probiotic (N=30)** | **Control (N=24)** | **Overall (N=54)** |
| --- | --- | --- | --- | --- |
|  |  | **n (%)** | **n (%)** | **n (%)** |
| Child lived in or regularly visited a building built before 1978 with recent or ongoing painting, repair, remodeling, or damage, in the last 6 months | Yes | 0 (0.0) | 4 (16.7) | 4 (7.4) |
|  | No | 30 (100.0) | 20 (83.3) | 50 (92.6) |
| Child has a family member or friend who has or had an elevated blood lead level | Yes | 1 (3.3) | 0 | 1 (1.9) |
|  | No | 29 (96.7) | 24 (100.0) | 53 (98.1) |
| Child frequently puts things in his/her mouth, such as toys, jewelry, or keys; child eat non-food items like paint chips or dirt | Yes | 17 (56.7) | 12 (50.0) | 29 (53.7) |
|  | No | 13 (43.3) | 12 (50.0) | 25 (46.3) |
| Child frequently comes in contact with an adult whose job or hobby involves exposure to lead | Yes | 1 (3.3) | 7 (29.2) | 8 (14.8) |
|  | No | 29 (96.7) | 17 (70.8) | 46 (85.2) |
| Child lives near an active lead smelter, battery recycling plant, or another industry likely to release lead, or near a heavily-traveled road | Yes | 3 (10.0) | 1 (4.2) | 4 (7.4) |
|  | No | 27 (90.0) | 23 (95.8) | 50 (92.6) |
| Child often comes into contact with traditional Chinese medicine and inferior/substandard cosmetics; uses leaded food utensils at home | Yes | 4 (13.3) | 4 (16.7) | 8 (14.8) |
|  | No | 26 (86.7) | 20 (83.3) | 46 (85.2) |
| Child often or ever uses unqualified colored toys or paint brushes | Yes | 4 (13.3) | 5 (20.8) | 9 (16.7) |
|  | No | 26 (86.7) | 19 (79.2) | 45 (83.3) |

Full Analysis Set population.

**Supplemental Table 2. Overview of Adverse Events (AEs)**

|  | **Probiotic (N= 30)** | | **Placebo (N= 24)** | | **Overall (N= 54)** | |
| --- | --- | --- | --- | --- | --- | --- |
| **Items** | **Events** | **Subjects (%)** | **Events** | **Subjects (%)** | **Events** | **Subjects (%)** |
| AEs | 33 | 17 (56.7) | 30 | 15 (62.5) | 63 | 32 (59.3) |
| TEAEs | 30 | 15 (50.0) | 26 | 14 (58.3) | 56 | 29 (53.7) |
| TRAEs | 0 | 0 | 0 | 0 | 0 | 0 |
| SAEs | 0 | 0 | 1 | 1 (4.2) | 1 | 1 (1.9) |
| SAEs Related to the Investigational Food | 0 | 0 | 0 | 0 | 0 | 0 |
| TEAEs Leading to Investigational Food Interruption | 1 | 1 (3.3) | 0 | 0 | 1 | 1 (1.9) |
| TRAEs Leading to Investigational Food Interruption | 0 | 0 | 0 | 0 | 0 | 0 |
| TEAEs Leading to Investigational Food Permanently Discontinued | 0 | 0 | 0 | 0 | 0 | 0 |
| TRAEs Leading to Investigational Food Permanently Discontinued | 0 | 0 | 0 | 0 | 0 | 0 |
| TEAEs Leading to Study Discontinuation | 0 | 0 | 0 | 0 | 0 | 0 |
| TRAEs Leading to Study Discontinuation | 0 | 0 | 0 | 0 | 0 | 0 |

Safety Set Population. Abbreviations: N= Number of subjects in the analysis set; TEAE= Treatment Emergent Adverse Event; TRAE= Treatment-related Adverse Event; SAE= Serious Adverse Event.

Note: A treatment-emergent adverse event (TEAE) is an AE that starts on/after the day that subjects receive the first study treatment.

Note: A treatment-related adverse event (TRAE) is an TEAE that is related to the investigational product.

Note: The causality between AEs and intervention was classified into five relationships: Definitely Related, Probably Related, Potentially Related, Unlikely to be related, and Not Related. All the definitely related, probably related, and potentially related adverse events will be considered related to the study intervention.

Note: Denominator of subjects (%) was N.

Note: Subject with SAE was diagnosed with acute tonsillitis.

Note: Subject with TEAEs Leading to Investigational Food Interruption was diagnosed with acute upper respiratory infection.

**Supplemental Table 3. Incidence and Number of Treatment-Emergent Adverse Events (TEAEs)**

|  | **Probiotic (N= 30)** | | **Placebo (N= 24)** | | **Overall (N= 54)** | |
| --- | --- | --- | --- | --- | --- | --- |
| **Adverse Event Term** | **Events** | **Subjects (%)** | **Events** | **Subjects (%)** | **Events** | **Subjects (%)** |
| All | 30 | 15 (50.0) | 26 | 14 (58.3) | 56 | 29 (53.7) |
| Upper respiratory tract infection | 12 | 8 (26.7) | 10 | 8 (33.3) | 22 | 16 (29.6) |
| Dyspepsia | 1 | 1 (3.3) | 5 | 2 (8.3) | 6 | 3 (5.6) |
| Pyrexia | 2 | 2 (6.7) | 1 | 1 (4.2) | 3 | 3 (5.6) |
| Rhinitis allergic | 2 | 2 (6.7) | 3 | 1 (4.2) | 5 | 3 (5.6) |
| Attention deficit hyperactivity disorder | 2 | 2 (6.7) | 0 | 0 | 2 | 2 (3.7) |
| Dermatitis allergic | 1 | 1 (3.3) | 1 | 1 (4.2) | 2 | 2 (3.7) |
| Pharyngitis | 1 | 1 (3.3) | 1 | 1 (4.2) | 2 | 2 (3.7) |
| Rhinitis | 3 | 2 (6.7) | 0 | 0 | 3 | 2 (3.7) |
| Vitamin D deficiency | 0 | 0 | 2 | 2 (8.3) | 2 | 2 (3.7) |
| Allergic cough | 1 | 1 (3.3) | 0 | 0 | 1 | 1 (1.9) |
| Anaemia | 0 | 0 | 1 | 1 (4.2) | 1 | 1 (1.9) |
| Balanoposthitis | 1 | 1 (3.3) | 0 | 0 | 1 | 1 (1.9) |
| Eczema | 1 | 1 (3.3) | 0 | 0 | 1 | 1 (1.9) |
| Infectious mononucleosis | 0 | 0 | 1 | 1 (4.2) | 1 | 1 (1.9) |
| Noninfective gingivitis | 1 | 1 (3.3) | 0 | 0 | 1 | 1 (1.9) |
| Provisional tic disorder | 1 | 1 (3.3) | 0 | 0 | 1 | 1 (1.9) |
| Swelling face | 1 | 1 (3.3) | 0 | 0 | 1 | 1 (1.9) |
| Tonsillitis* | 0 | 0 | 1 | 1 (4.2) | 1 | 1 (1.9) |
|  | | | | | | |

Safety Set Population. Abb: N= Number of subjects in the analysis set; TEAE= Treatment Emergent Adverse Event.

Note: A treatment-emergent adverse event (TEAE) is an AE that starts on/after the day that subjects receive the first study treatment.

Note: Denominator of subjects (%) was N.

Note: Tonsilitis was evaluated as serious AE but not related to the investigational product.

**Supplemental Table 4. Vital signs**

|  | **Probiotic** | | **Placebo** | |
| --- | --- | --- | --- | --- |
|  | N | **Mean (SD)** | **N** | **Mean (SD)** |
| **TEMPERATURE (°C)** | | | | |
| Baseline | 30 | 36.5 (0.2) | 24 | 36.4 (0.1) |
| Week 4 | 24 | 36.4 (0.2) | 22 | 36.4 (0.2) |
| Week 8 | 25 | 36.5 (0.2) | 21 | 36.5 (0.2) |
| Week 12 | 21 | 36.4 (0.2) | 18 | 36.5 (0.2) |
| **PULSE (BEATS/MIN)** | | | | |
| Baseline | 30 | 84.5 (11.7) | 24 | 82.5 (17.1) |
| Week 4 | 24 | 82.6 (11.1) | 22 | 83.4 (9.1) |
| Week 8 | 25 | 80.8 (9.4) | 21 | 79.1 (16.6) |
| Week 12 | 21 | 79.3 (8.8) | 18 | 85.6 (10.1) |
| **BREATHING (BREATHS/MIN)** | | | | |
| Baseline | 30 | 22.6 (3.4) | 24 | 24.8 (14.1) |
| Week 4 | 24 | 21.1 (2.1) | 22 | 25.3 (18.2) |
| Week 8 | 25 | 24.2 (17.0) | 21 | 23.8 (12.6) |
| Week 12 | 21 | 20.6 (1.9) | 18 | 21.0 (2.4) |
| **SYSTOLIC BLOOD PRESSURE (mmHg)** | | | | |
| Baseline | 30 | 103.0 (13.8) | 24 | 99.4 (9.8) |
| Week 4 | 24 | 101.4 (11.0) | 22 | 97.7 (12.3) |
| Week 8 | 25 | 99.3 (11.9) | 21 | 101.8 (9.2) |
| Week 12 | 21 | 102.6 (11.4) | 18 | 98.4 (8.3) |
| **DIASTOLIC BLOOD PRESSURE (mmHg)** | | | | |
| Baseline | 30 | 62.9 (8.0) | 24 | 57.6 (5.6) |
| Week 4 | 24 | 62.4 (4.4) | 22 | 58.1 (9.8) |
| Week 8 | 25 | 63.2 (11.9) | 21 | 63.8 (7.2) |
| Week 12 | 21 | 61.9 (5.0) | 18 | 60.4 (5.9) |

Safety Set Population. Abbreviations: N= Number of subjects in the analysis set; n= Number of subjects providing data in the treatment cohort; SD= Standard Deviation; Min= Minimum; Max= Maximum.

Note: Only subjects who had assessment records at baseline and had at least on assessment record at post-baseline visits for each test were included in the summary.
